# Effects of season and food on the scatter-hoarding behavior of rodents in temperate forests of Northeast China

**DOI:** 10.3897/zookeys.1025.60972

**Published:** 2021-03-18

**Authors:** Dianwei Li, Yang Liu, Hongjia Shan, Na Li, Jingwei Hao, Binbin Yang, Ting Peng, Zhimin Jin

**Affiliations:** 1 College of Life Sciences and Technology, Mudanjiang Normal University, No. 191 Wenhua Road, Mudanjiang, Heilongjiang 157011, China Mudanjiang Normal University Mudanjiang China; 2 College of Wildlife and Protected Area, Northeast Forestry University, No. 26 Hexing Road, Harbin 150040, China Northeast Forestry University Harbin China; 3 Heilongjiang Academy of Forestry, No. 134 Haping Road, Harbin, Heilongjiang 150081, China Heilongjiang Academy of Forestry Harbin China; 4 Mudanjiang Medical School No. 5 Fangzhier Road, Mudanjiang, Heilongjiang 157009, China Mudanjiang Medical School Mudanjiang China

**Keywords:** Murids, rodents, scatter-hoarding, seed fate, seed dispersal, voles

## Abstract

To explore the differences in hoarding strategies of rodents for different seeds in various seasons, we labeled and released the seeds of *Pinus koraiensis*, *Corylus mandshurica*, *Quercus mongolica* and *Prunus sibirica* in temperate forests of Northeast China and investigated the fate of the seeds in spring and autumn. The analysis showed that the hoarding strategies of the rodents varied substantially between seasons. The seeds were consumed faster in the spring than in the autumn. More than 50% of the seeds in the two seasons were consumed by the 16^th^ day. It took 36 days to consume 75% of the seeds in the spring and 44 days in the autumn. The rate of consumption of the seeds in the spring was greater than in the autumn, and the rate of spread of the seeds was greater in the autumn. The distances of removal for the consumption and dispersal of seeds in the spring (3.26 ± 3.21 m and 4.15 ± 3.52 m, respectively) were both shorter than those in the autumn (3.74 ± 3.41 m and 4.87 ± 3.94 m, respectively). In addition, the fate of different seeds varied significantly owing to differences in hoarding strategies. The seeds of the three preferred species, *P. koraiensis*, *C. mandshurica*, and *Q. mongolica*, were quickly consumed. More than 90% of the seeds of these species were consumed. Only 21% of *Pr. sibirica* seeds were slowly consumed, and the two seasons had the same seed consumption rate patterns: the consumption rate of *P. koraiensis* seeds was the highest, followed by *C. mandshurica*, then *Q. mongolica*, and finally *Pr. sibirica*. The median removal times of the two seasons were different, but the rules were the same: *P. koraiensis* was the shortest, followed by *C. mandshurica*, and the third was *Q. mongolica*. In both seasons, the most predated *in situ* seeds were those of *P. koraiensis*; the most hoarded seeds were those of *C. mandshurica*, and the most unconsumed seeds were those of *Pr. sibirica*.

## Introduction

Rodents play an important role in the process of plant seed dispersal and regeneration (Hughes and Croy 1993; Vander Wall 2001; Vander Wall 2003; Lichti et al. 2015). Seed dispersal is a key link that affects plant regeneration, species survival and distribution (Vander Wall 2001; Vander Wall 2003; Lichti et al. 2015), and plays a vital role in forest ecosystems. The carrier behavior of animals determines the pattern of seed dispersal, which fundamentally affects the dynamics of vegetation populations and communities, and even forest ecosystems (Vander Wall 1990; Clayton et al. 2001; Vander Wall 2001; Briggs et al. 2009; Lichti et al. 2015). Rodents often dominate the local dynamics of forest regeneration, since they are the main scatter dispersers of plant seeds (Morán-López et al. 2016; Morán-López et al. 2018). This results in their role as an important seed disperser in forest ecosystems (Morán-López et al. 2015; Morán-López et al. 2018).

The hoarding behavior of rodents is a special type of feeding behavior; it is a vital adaptive strategy for many rodents during periods of food scarcity (Chang et al. 2010; Luna et al. 2016; Li et al. 2018, 2020). The hoarding behavior is affected by many factors, including the characteristics of plant seeds, yield, distribution, temporal and spatial changes of food resources, as well as seasonal changes in environmental factors, such as climate and habitat structure (Willson and Whelan 1990; Vander Wall 2001; Preston and Jacobs 2005; Jenkins 2011; Lichti et al. 2015; Morán-López et al. 2018; Li et al. 2018). The community structure and the quantity of rodents display obvious seasonal fluctuations in temperate regions, and the intensity of food hoarding activities also varies significantly in different seasons of the year (Vander Wall 1990). A reasonable allocation of limited food resources, an adjustment of the temporal and spatial abundance, and the use pattern of food help animals utilize hoarded resources to ensure their survival or reproductive activities during food shortages (Smith and Reichman 1984; Vander Wall 2001; Lu and Zhang 2005; Chang et al. 2010; Wang et al. 2012; Lichti et al. 2015; Luna et al. 2016). Food resources fluctuate greatly, particularly in the Northeast China in which the seasonal changes in the habitat environment are significant; the climatic environment changes are significantly, and the habitat conditions are unstable (Li et al. 2019 and 2020). Hoarding food is an important way to manage severe environments, effectively save predation time and energy consumption, and ensure that the rodents successfully survive the winter (Zhang et al. 2014; Li et al. 2018, 2020).

The Northeast China temperate zone is rich in forest vegetation resources and is an important resource of species and bank for seeds. Rodents not only damage forest resources by their predation on vegetation and seeds, but also promote the regeneration of vegetation by scatter-hoarding food (Vander Wall 2001; Vander Wall 2003; Lichti et al. 2015). *Pinus koraiensis* (Pinaceae), *Corylus mandshurica* (Betulaceae), *Quercus mongolica* (Fagaceae) and *Prunus sibirica* (Rosaceae) are common forest trees in the study area, and their seeds are the main food resources for rodents. In this study, four types of labeled seeds were released in the spring and autumn to investigate the predation, dispersal, and hoarding of seeds by rodents to understand the patterns of utilization of various seeds by rodents and the existing seasonal laws in the natural environment. Our goals were to provide theoretical and practical guidance to explore the interaction between rodents and many large-seed plants. We hypothesized the following: i) Because of the lack of food resources in the spring and abundance of seeds in the autumn, there are seasonal differences in seed dispersal, and seeds released in the spring will be consumed more quickly and in greater quantities than those released in the autumn; ii) to meet the different needs, more food is taken in the spring, and the amounts of dispersal and distance are greater in the autumn; and iii) rodents have predation preferences, and different seeds have various fates with the preferred seeds being consumed faster.

## Site and methods

### Study area and research site selection

The study was conducted from April to November, 2018. The research site was located in a forested area of the Sandao Forest Farm (44°40'N–44°45'N, 129°24'E–129°32'E, elevation 380 to 550 m), Mudanjiang City, located at the northern end of the Changbai Mountains in northeastern China, the east vein of the main ridge of the Zhangguangcai Mountain. The climate is a temperate and cold continental monsoon climate with four distinct seasons and a hot rainy season. The maximum temperature is 37 °C. The minimum temperature is -44.1 °C, and the annual average temperature is 2.3–3.7 °C. Approximately 100–160 days in the year are frost free. The first frost in most areas appears is in late September, and the last frost is in late April to early May. Precipitation is concentrated in June to September and varies from 400 mm to 800 mm.

The experiment was conducted in secondary coniferous and broad-leaved mixed forest that had been least disturbed. Common canopy tree species included *P. koraiensis*, *Q. mongolica*, *Larix gmelinii*, *Picea koraiensis*, *Abies nephrolepis*, *Betula platyphylla*, *Tilia amurensis*, *T. mandshurica*, *Juglans mandshurica*, while the brush included primarily *C. mandshurica*, *Lonicera maackii*, *Acanthopanax senticosus*, *Trichosanthes kirilowii*, and *Syringa reticulata*. The rodents in forests were highly abundant and diverse, largely dominated by different combinations of species, with *Apodemus peninsulae*, *A. agrarius*, and *Clethrionomys rufocanus* being the dominant species.

### Tagging and tracking of seeds

Healthy seeds of *P. koraiensis*, *C. mandshurica*, *Q. mongolica*, and *Pr. sibirica* that had been selected in the field study experiment were tagged using an electric drill whose bit is 0.5 mm in diameter, to make a hole at one end of the seed. A thin red plastic sheet was cut into a 3 cm × 1 cm rectangular plastic plate (Xiao et al. 2006a; Hirsch et al. 2013), and a small hole was introduced into the middle of short side. A soft steel wire, 0.3 mm in diameter and 8 cm long, was used to connect the perforated seeds to the plastic plate and mark the seed category, sample number, and seed number on each tag. Based on experiments, after rodents ate the seeds or buried them in the ground, under dead branches, or in shallow caves, the tags would be exposed, which were convenient for positioning during studies. Rodents could not bite off the steel wire; therefore, this tagging method had no significant impact on their seed dispersal (Xiao et al. 2006b; Chang and Zhang 2011; Vander Wall and Beck 2011; Yi et al. 2012; Luna et al. 2016; Li et al. 2018)

### Release and investigation of the seeds

Food release stations in the forest were randomly arranged and spaced more than 50 m apart. Each release station released 20 seeds of each type for a total of 80. There were 15 release stations in the spring with 300 seeds of each type, totaling 1200. There were 9 release stations in the autumn with 180 seeds of each type, totaling 720.

The study was conducted on days 1, 2, 3, 4, 6, 8, 12, 16, 20, 28, 36, 44, and 60 after release. The fate and characteristics and dispersal distance of the seeds were measured.

### Definition of seed fate

The fate of the seeds released in the field experiment is defined as follows (Li and Zhang 2003; Pons and Pausas 2006; Yang et al. 2011; Li et al. 2018):

1. Intact *in situ* (IS): seeds not eaten or removed from the station

2. Predation *in situ* (PS): seed kernels eaten at the seed station

3. Predation after removal (PR): seed kernels eaten after removal

4. Intact after removal (IR): seeds not eaten and abandoned on the surface of ground after removal

5. Hoarded after removal (HR): seeds buried in the soil or humus layer after removal

6. Missing after removal (MR): seeds removed but not found

7. Consumption: With the exception of IS seeds, the fate of other seeds is defined as consumption by rodents.

8. Predation: PS and PR are defined as predation (PS + PR)

9. Dispersal: IR, HR, and MR are defined as dispersal. However, there were no data records for the survey indicators of the missing seeds, so they could not be calculated during the inspection and comparison (IR + HR + MR)

10. Median removal time (MRT) of the seeds: the time at which 50% of the seeds were removed (expressed in days), which was used to compare the seed removal rates in both types of vegetation.

### Statistical analysis

All statistical analyses were conducted in SPSS 22.0 for Windows (IBM, Inc., Armonk, NY, USA). Before the data analysis, the data was tested for normality and equality of variance using the Kolmogorov-Smirnov and Homogeneity-of-variance tests. Data were treated with respective nonparametric tests depending on whether they met the assumptions of normality. A Cox regression was used to analyze the seed survival rates, factoring in both seasons’ type and seeds. The Kruskal–Wallis H test (nonparametric) was used to compare the significant differences among the four seed species. The Mann–Whitney U test (nonparametric) was used to test the differences between the different seasons and different seed species. The data are represented as the mean ± SD. The values are considered statistically significant at *P* < 0.05.

## Results

### Seed survival curves

**Figure 1.**
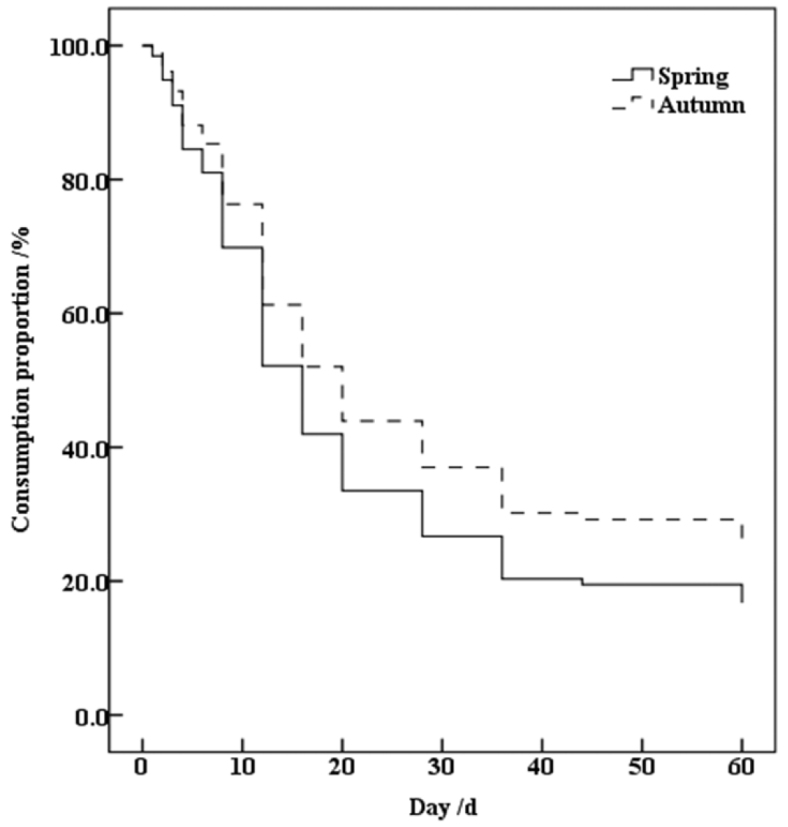
The total survival curve of the all seeds of *Pinus koraiensis*, *Corylus mandshurica*, *Quercus mongolica* and *Prunus sibirica* in the spring and autumn in temperate forests of Northeast China.

According to the analysis of the seed survival curve (Fig. 1), the early seeds were mostly consumed quickly. And in general, the rate of consumption gradually decreased after the 20^th^ day. Most seeds (*P. koraiensis*, *C. mandshurica*, and *Q. mongolica*) preferred by rodents were depleted over time, and most of the seeds not favored (*Pr. sibirica*) were IS. Therefore, when the curve survival ratio approached 20%, the curve flattened, and the trend of seed consumption became smaller.

According to the analysis of seed survival curves in different seasons (Fig. 1), the trends of consumption of seeds in the spring and autumn were the same, but the rates of consumption were different. The rate of consumption in the spring was faster than that in the autumn (*W* = 32.395, df = 1, *P* < 0.001). Most of the seeds of *Pr. sibirica* were not consumed, and the trend of consumption gradually slowed at approximately 20% of the curve.

**Figure 2.**
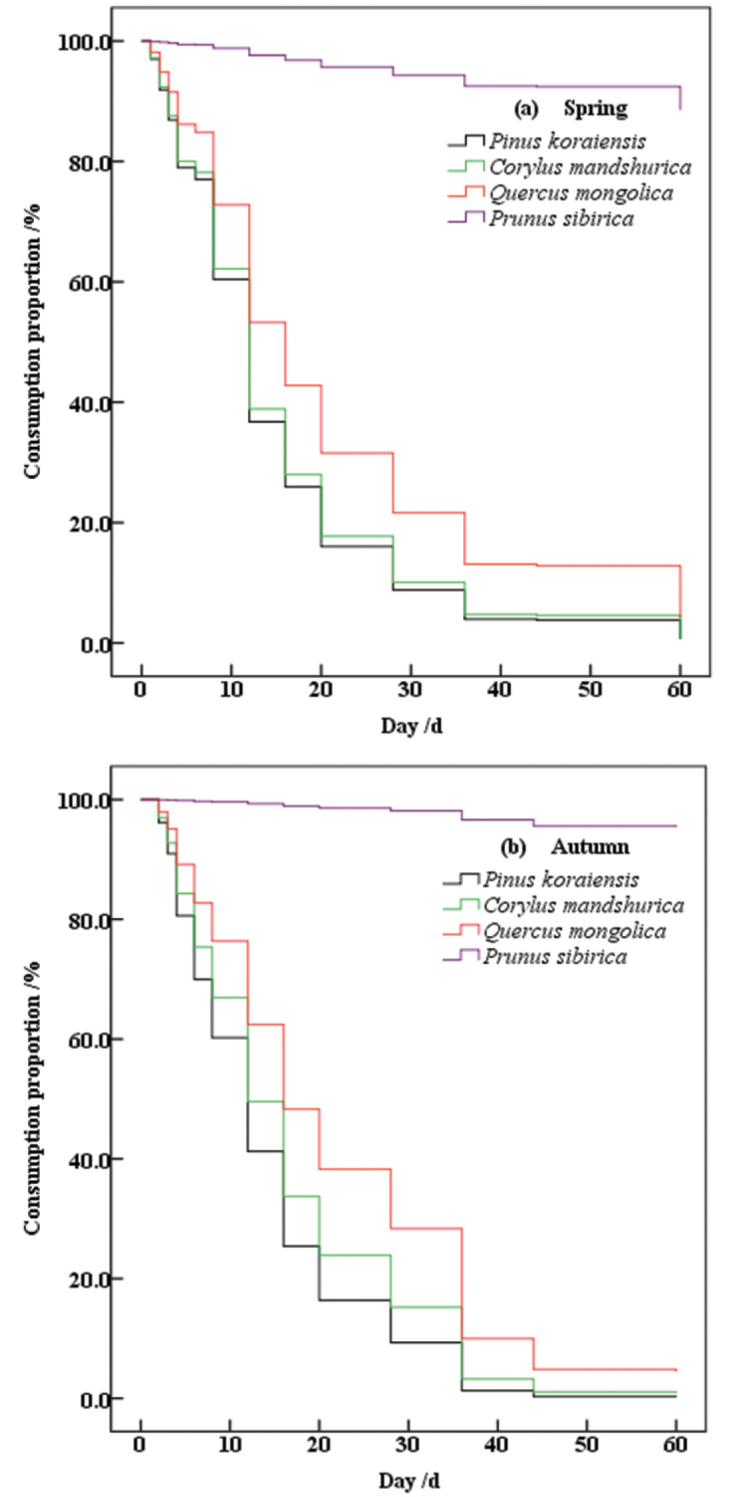
The survival curve of the four types of seeds of *Pinus koraiensis*, *Corylus mandshurica*, *Quercus mongolica* and *Prunus sibirica* respectively in spring and autumn in temperate forests of Northeast China

According to the analysis of survival curves of four types of seeds in the spring and autumn (Fig. 2), the consumption rates of different seeds varied. The three types of seeds from *P. koraiensis*, *C. mandshurica*, and *Q. mongolica* had similar trends of consumption, but the rates of consumption were different (Spring: *W* = 43.215, df = 2, *P* < 0.001; Autumn: *W* = 31.710, df = 2, *P* < 0.001), with *Pr. sibirica* seed consumption being slow. The consumption rate laws of the four types of seeds in the two seasons is as follows. The consumption rate of *P. koraiensis* was the highest, followed by *C. mandshurica*, then *Q. mongolica*, and finally *Pr. sibirica*. (Spring: *W* = 259.542, df = 3, *P* < 0.001; Autumn: *W* = 114.104, df = 3, *P* < 0.001).

### Seed consumption time of the rodents

There were no differences in the average time of the earliest food discovery between the two seasons (*Z* = -0.508, *P* > 0.05). After the animals found the seeds, they chose different ones based on their preferences and performed different operations, resulting in various fates of the seeds. The sequences of the earliest consumption time of seeds in the spring and autumn were both as follows: *C. mandshurica* was the earliest, followed by *P. koraiensis*, then *Q. mongolica*, and finally *Pr. sibirica*. There were no differences in the earliest consumption time among *P. koraiensis*, *C. mandshurica*, and *Q. mongolica* in different seasons (spring: *χ^2^* = 1.140, *P* > 0.05; autumn: *χ^2^* = 0.634, *P* > 0.05). The earliest time of consumption of *Pr. sibirica* was significantly later than that of the other types of seeds in the spring and autumn (all *P* < 0.05) (Table 1) .

**Table 1.** The earliest discovery time and consumption time of the different seeds in spring and autumn in temperate forests of Northeast China. The different superscript letters represent significant differences from each other (*P* < 0.05), based on Mann–Whitney U testing differences in the earliest discovery time between the spring and autumn, based on Kruskal–Wallis H testing differences in the earliest consumption time among the four seed species.

Season	Earliest discovery time (range; day)	Earliest consumption time (range; day)			
		*P. koraiensis*	*C. mandshurica*	*Q. mongolica*	*Pr. sibirica*
Spring	7.2 ± 8.8 (1–36)^a^	10.7 ± 13.8 (1–60^)^b	9.4 ± 13.0 (1–60)^b^	9.6 ± 7.9 (1–^6^0)b	75.4 ± 38.1 (6–108^)^c
Autumn	5.8 ± 3.8 (2–12)^a^	10.3 ± 8.1 (2–28^)^b	9.1 ± 7.87 (3–28)^b^	11.1 ± 7.0 (4–20)^b^	70.7 ± 33.9 (12–92)^c^

### Median removal time (MRT) of the seeds

When the seed species were not distinguished, the MRT was around the 16^th^ day. Seventy percent of the seeds were consumed at approximately the 36^th^ day, and 18.08% of the seeds were still unconsumed after the 90^th^ day (Fig. 1). *Pr. sibirica* comprised 90.46% of the unconsumed seeds. A statistical analysis of the data (Table 2, Fig. 2) indicated that the MRT was on the 16^th^ day in the spring and autumn, and it took time for 75% of the seeds to be consumed. This did not happen until the 36^th^ day in spring and the 44^th^ day in autumn. However, the differences in MRT between spring and autumn were nonsignificant (*Z* = -0.074, *P* > 0.05). The times for 50% of the seeds to be consumed varied for different seeds. Spring and autumn showed the same pattern: *P. koraiensis* had the shortest time, followed by *C. mandshurica*, while *Q. mongolica* had the longest time. However, there was no significant difference between the three types of seeds (Spring: *χ^2^* = 4.480, *P* > 0.05; Autumn: *χ^2^* = 3.089, *P* > 0.05). The rates of consumption of the *Pr. sibirica* seeds did not exceed 50% during the spring and autumn studies. The autumn study led to the discovery that some of the *Pr. sibirica* seeds released in the spring remained at the seed station. The consumption curve estimated that the time when 50% of the *Pr. sibirica* seeds were consumed should exceed 150 d (Fig. 2).

**Table 2.** Median time of removal of the different seeds in spring and autumn in temperate forests of Northeast China. The same superscript letters represent nonsignificance (*P* > 0.05), based on Mann–Whitney U testing differences in MRT between the spring and autumn, based on Kruskal–Wallis H testing differences in MRT among the three seed species.

Season	Median removal time (day)			
	*P. koraiensis*	*C. mandshurica*	*Q. mongolica*	*Pr. sibirica*
Spring	13.6 ± 12.^9^a	14.4 ± 14.0^a^	18.5 ± 10.1^a^	–
Autumn	10.3 ± 8.1^a^	13.8 ± 7.0^a^	18.7 ± 12.4^a^	–

### Seed removal time

Less than 100% of the seeds were consumed during the study. Therefore, the average removal time of the three types of seeds (*P. koraiensis*, *C. mandshurica* and *Q. mongolica*) was estimated based on the survey data of the seed station after predation (Table 3). The latest time of *Q. mongolica* removal was significantly later than that of *P. koraiensis* in the spring (*Z* = -2.283, *P* < 0.05), while there was no difference for *C. mandshurica* (*Z* = -1.395, *P* > 0.05). The latest time of *Q. mongolica* removal was significantly later than those of *P. koraiensis* (*Z* = -3.446, *P* < 0.001) and *C. mandshurica* (*Z* = -2.650, *P* < 0.01) in the autumn.

**Table 3.** The latest consumption time of the different seeds in spring and autumn in temperate forests of Northeast China. The different superscript letters represent significant differences from each other (*P* < 0.05), based on Mann–Whitney U testing differences in the latest consumption time between the spring and autumn, based on Kruskal–Wallis H testing differences in the latest consumption time among the three seed species.

Season	The latest consumption time (range; day)			
	*P. koraiensis*	*C. mandshurica*	*Q. mongolica*	*Pr. sibirica*
Spring	18.3 ± 12.5(2–60)^a^	22.1 ± 17.0(4–76)^ab^	28.5 ± 17.7(12–76)^b^	–
Autumn	17.3 ± 7.2(8–28)^a^	24.0 ± 12.8(4–44)^a^	46.7 ± 17.9(28–76)^b^	–

### Seed fate

**Figure 3.**
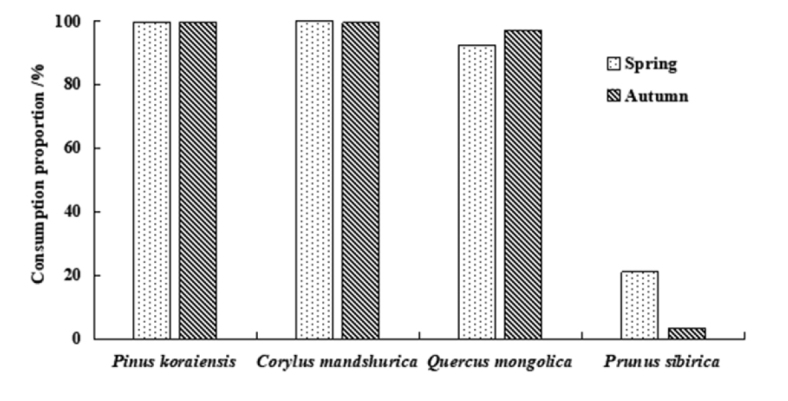
The consumption proportion of the four types of seeds in spring and autumn in temperate forests of Northeast China.

Different seeds had varied fates, and the seasonal differences in the seed fates were significant. More seeds were consumed in the spring than in the autumn. With the exception of *Pr. sibirica* seeds, the rates of consumption of the seeds of the other three species all exceeded 90%. In the spring, the rate of consumption of *P. koraiensis* seeds reached 99.56%, *C. mandshurica* seeds 100%, and *Q. mongolica* seeds 92.35%. In the autumn, the rate of consumption of *P. koraiensis* seeds reached 99.44%, *C. mandshurica* seeds 99.44%, and *Q. mongolica* seeds 97.22%. The rates of consumption of *Pr. sibirica* seeds in the spring and autumn were 21.0% and 3.33%, respectively (Fig. 3)

**Figure 4.**
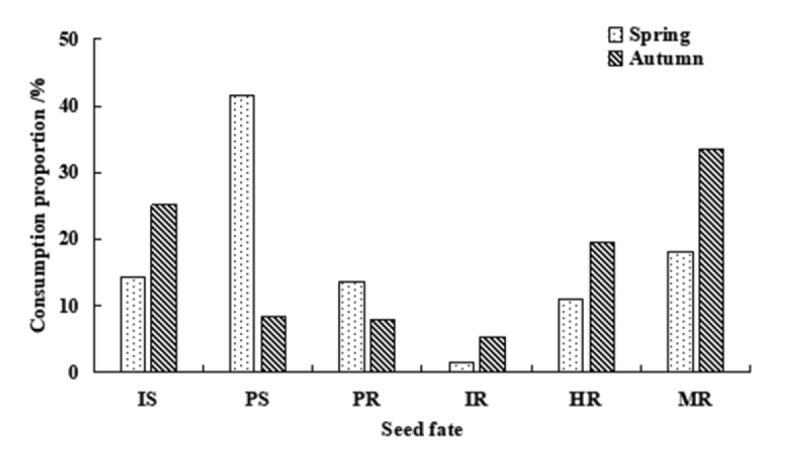
Statistics of the fate of seeds in the spring and autumn in temperate forests of Northeast China. Abbreviations: IS–Intact *in situ*, PS–Predation *in situ*, PR–Predation after removal, IR–Intact after removal, HR–Hoarded after removal, MR–Missing after removal.

**Figure 5.**
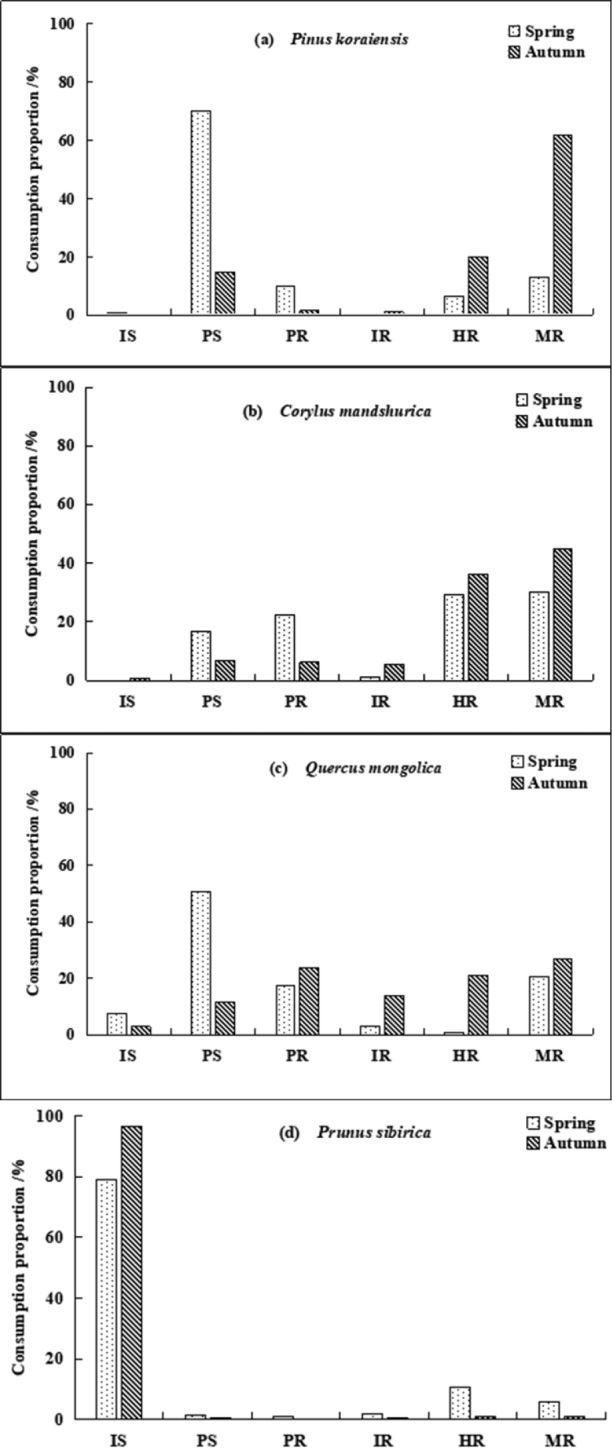
Statistics on the fate of the four types of seeds combined in spring and autumn in temperate forests of Northeast China. Abbreviations: IS–Intact *in situ*, PS–Predation *in situ*, PR–Predation after removal, IR–Intact after removal, HR–Hoarded after removal, MR–Missing after removal.

In the spring, the proportions of seeds that were intact *in situ* (IS), predation *in situ* (PS), predation after removal (PR), intact after removal (IR), hoarded after removal (HR), and missing after removal (MR) were 14.20%, 41.52%, 13.59%, 1.45%, 11.07% and 18.17%, respectively. In the autumn, the proportions of seeds with corresponding fates were 25.14%, 8.47%, 7.92%, 5.28%, 19.58% and 33.61%, respectively (Fig. 4). The unconsumed seeds were primarily *Pr. sibirica* (comprising 84.95% in the spring and 96.13% in the autumn). *P. koraiensis* accounted for the most predation *in situ* seeds (57.90% in the spring and 44.26% in the autumn), followed by *Q. mongolica* (31.62% in the spring and 34.43% in the autumn). *C. mandshurica* had the largest proportion among the hoarded seeds (64.17% in the spring and 46.10% in the autumn) (Fig. 5).

**Figure 6.**
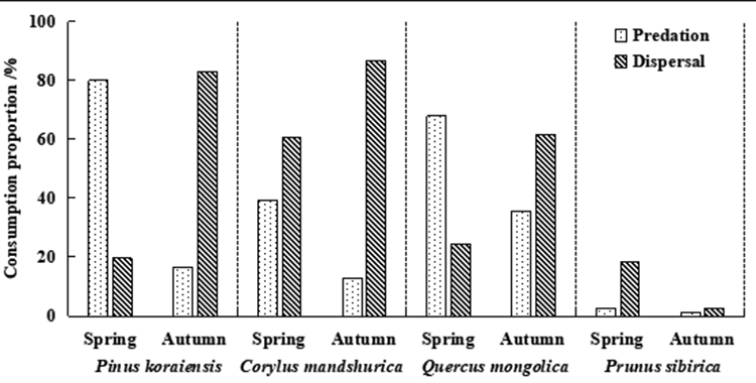
Predation rate and dispersal rate of four kinds of seeds combined in spring and autumn in temperate forests of Northeast China.

The total predation rate of seeds was higher in the spring (55.11%) than in the autumn (16.39%). The total seed dispersal rate of seeds was higher in the autumn (58.47%) than in the spring (30.69%) (Fig. 5). This pattern was also evident in the three types of seeds of *P. koraiensis*, *C. mandshurica*, and *Q. mongolica*. In the spring, the predation rate of *P. koraiensis*, *C. mandshurica*, and *Q. mongolica* was 80.0%, 30.38%, and 67.94%, respectively, and the dispersal rate was 19.56%, 60.32%, and 24.41%, respectively. In the autumn, the predation rate of *P. koraiensis*, *C. mandshurica*, and *Q. mongolica* was 16.67%, 12.77%, and 35.56%, respectively, and the dispersal rate was 82.77%, 86.67%, and 61.66%, respectively (Fig. 6).

### Transport distance of predation after removal

The transport distances of seeds by PR in the spring and autumn were 3.26 ± 3.21 m and 3.74 ± 3.41 m, respectively, with no significant difference (*Z* = -1.276, *P* = 0.202 > 0.05).

The three species of seeds *P. koraiensis* (3.51 ± 3.25 m in the spring, 3.30 ± 2.03 m in the autumn), *C. mandshurica* (3.17 ± 2.91 m in the spring, 3.88 ± 2.19 m in the autumn), *Q. mongolica* (2.72 ± 3.37 m in the spring, 3.74 ± 3.76 m in the autumn) showed no seasonal differences in the transport distances of PR. *Pr. sibirica* seeds (7.73 ± 3.36 m in the spring) showed no PR of data records during the autumn study.

### Dispersal distance

The seed dispersal distances in the spring and autumn were 4.15 ± 3.52 m and 4.87 ± 3.94 m, respectively, and the dispersal distance in the spring was significantly shorter than that in the autumn (*Z* = -2.008, *P* < 0.05). The dispersal distance of *P. koraiensis* seeds was significantly greater than that of the other three types (*χ^2^* = 24.975, *P* < 0.001), and there was a difference between the other three types (all *P* > 0.05). The dispersal distance of *P. koraiensis* seeds in the spring (3.89 ± 2.05 m) was significantly shorter than that in the autumn (7.97 ± 5.33 m) (*Z* = -3.762, *P* < 0.001). *C. mandshurica* seeds (4.42 ± 3.72 m in the spring, and 4.12 ± 2.49 m in the autumn) , *Q. mongolica* seeds (6.16 ± 6.27 m in the spring, 3.69 ± 3.31 m in the autumn) and *Pr. sibirica* seeds (2.29 ± 1.50 m in the spring, 3.18 ± 2.66 m in the autumn) showed no seasonal differences in the diffusion distances (all *P* > 0.05). The dispersal distances of seeds were greater than the transport for predation distances (all *P* < 0.05).

## Discussion

### Seasonal differences in the food hoarding strategies of rodents

Some studies have shown that feeding and hoarding behavior of rodents had significant seasonal differences and were seen as an adaptation strategy in response to seasonal changes in food and the environment in Northeast China (Li et al. 2020). Our results about survival curves, consumption time, MRT, removal time and fate of seeds also indicated that the hoarding strategies of rodents varied substantially between seasons. For example, seeds were consumed faster and the rate of consumption of seeds was greater in the spring than in the autumn, however, the rate of spread of seeds was greater in the autumn than in the spring. The availability of food resources serves as the core factor that affects predation strategy, so dynamic hoarding strategy is an adaptation to changes in food resources and food quality (Vander Wall 1995; Vander Wall 2001; Chang et al. 2010; Lichti et al. 2015). Consequently, the rodent’s own demands show seasonal changes.

In response to the seasonal changes and the impacts on food resources, coupled with the north temperate climate, some rodents showed different requirements to meet different life activities at different times (Li et al. 2018). In the spring, the seed intake is greater, and the food is consumed faster, reflecting the crucial importance of obtaining food resources as soon as possible to supplement energy in the spring. This type of countermeasure is an important guarantee for survival, so that the rodents can readily enter the summer season with abundant resources and a favorable environment. In the autumn, food abundance weakens the predation strategy, but the dispersal is greater, indicating that more food must be hoarded to survive the winter successfully. These are the adaptation strategies of animals to the seasonal changes in food and environment, which are meaningful for their current and future survival and reproduction (Vander Wall 1990; Vander Wall 2001; Murray et al. 2006; Chang et al. 2010; Lichti et al. 2015).

Earlier results indicated that environment temperature has an important effect on changes in the activity behavior in rodents, with rodent species adjusting their foraging times and activities in response to changes in temperature (Corp et al. 1997; Li et al. 2020). The temperature is similar in the spring (April–May, 1–21°C) and autumn (September–October, 0–21°C) in the research area in 2018. However, the trend of temperature change is the opposite. Seasonal temperature changes send a strong signal to rodents, inducing different predation and hoarding behavior strategies (Corp et al. 1997; Li et al. 2020). The temperature gradually increased after April, rodents began spending more time on feeding activities. On the contrary, when temperatures began to cool after September, some rodents began spending more time on scatter-hoarding activities, suggesting that they were storing food in order to successfully overwinter (Vander Wall 2001; Chang et al. 2010; Lichti et al. 2015; Li et al. 2020).

### Differences in the selection of seeds by rodents

The differences in seed characteristics and the animal’s needs will affect the hoarding behavior of animals, which manifests as preference for food selection (Takahashi and Shimada 2008; Xiao et al. 2009; Ribeiro and Vieira 2016). Small animals have higher requirements for food quality, and food selectivity is more obvious (Fortin et al. 2004; Courant and Fortin 2012). The selection preference of rodents for seeds is the result of co–evolution between animals and plants. Seeds attract animals through morphological characteristics and nutritional components and induce animals to predate on, disperse, or hoard them, forming a mutually beneficial relationship (Vander Wall 1990; Vander Wall 2001; Chang et al. 2010; Zhang et al. 2014; Lichti et al. 2015). The four types of seeds used in the experiment induced differences in the predation and hoarding strategies of the rodents, such as more predation on *P. koraiensis* and *Q. mongolica* seeds, the more hoarding of *C. mandshurica* seeds, and a lack of selection of most *Pr. sibirica* seeds (Li et al. 2019). According to the Handling Costs hypothesis (Vander Wall 2010; Vander Wall and Beck 2011), seeds of *Q. mongolica* and *P. koraiensis*, which have higher contents of starch and fat, are prioritized to obtain the greatest energy benefits (Wang and Chen 2012; Li et al. 2018). *C. mandshurica* also has a high fat content, but the seed shell is thick and hard; the processing takes longer, and the cost, and risk of predation will increase. Therefore, hoarding is the optimal strategy. Moreover, *C. mandshurica* does not easily become moldy or be infested by moths, and it is more suitable for long-term hoarding than *Q. mongolica* (Vander Wall 2001; Yu et al. 2001). Therefore, the amount of *C. mandshurica* dispersed is the highest (60.32% in the spring, 86.67% in the autumn) in both the spring and autumn. *Pr. sibirica* has a small amount of predation and dispersal, indicating that there are some key factors that cause animal rejection. We hypothesize that it may be caused by the amygdalin compounds contained in almonds. This hypothesis requires an in–depth study. Different seeds vary in their diffusion distances, which is another type of selection difference exhibited by the rodents for different seed characteristics.

## Conclusions

Rodents change their predation and hoarding behavior strategies based on different seasons and foods. These animals adopt different foraging strategies so that the consumption of seeds has obvious seasonal differences. Food resources are scarce in the spring, and rodents spend more time foraging than in the autumn. Spring seeds are consumed in greater numbers and more quickly than in the autumn. In the spring, to meet the immediate demand for a supply of energy, rodents predate on more seeds. In the autumn, the abundance of seeds increases. To ensure the necessary food demand for winter survival, rodents disperse more seeds to different locations for hoarding, and the dispersal distance is relatively larger. For different seeds distributed in the same region, rodents will identify and judge the characteristics of the seeds, resulting in various fates for the seeds. Handling seeds in different ways leads to obvious predation preferences, and usually seeds that are rich in nutrients and easily handled are prioritized. Seeds with hard and thick seed coats are hoarded the most intensively.
